# Opinion: No

**DOI:** 10.1590/S1677-5538.IBJU.2015.06.04

**Published:** 2015

**Authors:** Kelly A. Chiles, Peter N. Schlegel

**Affiliations:** 1 Department of Urology, Brady Urology Foundation, Weill Cornell Medical College, New York, NY, USA

**Keywords:** Vasovasostomy, Sterilization Reversal, Infertility, Azoospermia, Fertility

The evaluation of any couple with infertility for any reason, including known post-vasectomy obstructive azoospermia, involves simultaneous evaluation of the female for her fertility potential with male evaluation. The goal of vasectomy reversal is to allow a couple to have a healthy child. In this overview, we will clarify that sperm retrieval with IVF is the most effective means for a couple to achieve their goal of having at least one healthy child. The limiting factor for natural conception and live birth after vasectomy is not typically the male partner's obstructive azoospermia, however, it is directly related to the female partner's fertility. The Practice Committee of the American Society for Reproductive Medicine clearly state in their guideline for management of obstructive azoospermia using reconstruction: “Before vasectomy reversal is performed to restore fertility, evaluation of the female partner's reproductive potential is prudent and recommended…” (1).

Women have a decline in reproductive potential that becomes significant by age 32-35 with a subsequent rapid decline (2). Age is not the only reason for impaired female fertility, and there is no age below which normal female reproductive potential can be guaranteed. Hormonal and congenital abnormalities as well as endometriosis are a few examples of common issues that greatly decrease the chance a couple has for a successful natural conception; all can be issues for women of any age. The observation that men are less likely to have a pregnancy with a new partner after vasectomy reversal than with same female partners, emphasize the role that the female plays in success rates of vasectomy reversal (3). These increased risks underscore the importance of the female evaluation because her fertility potential will frame the conversation regarding the risks, benefits, and likely outcomes of all reproductive possibilities, including natural conception.

Vasectomy reversal is an option for couples interested in fertility after vasectomy reversal. Vasectomy reversal has been reported to have some cost-benefits related to use of ART (4, 5). However, the “costs” associated with ART are overestimated by low pregnancy rates in historical published literature and excessive frequency of multiple gestations. Original cost-effectiveness studies with vasectomy reversal assumed a pregnancy rate nearly half the current pregnancy rate achieved with surgically retrieved sperm. In addition, the frequency of multiple gestations has been dramatically decreased by single embryo transfer and other contemporary enhancements in IVF. These changes dramatically improve the cost-effectiveness of sperm retrieval and IVF. Both treatment with both vasectomy reversal as well as sperm retrieval and IVF mandates that the male partner will undergo a procedure, the extent of the procedure with vasectomy reversal is far more extensive, requiring hours of anesthesia, typically general anesthesia, with its associated risks. The question at hand is not whether vasectomy reversal is a procedure that should be offered to couples. The question is whether it should be the first step for these couples. Every advantage that a vasectomy reversal provides couples relies on the female partner having normal fertility potential; therefore, a vasectomy reversal should only be performed after female reproductive capacity is confirmed.

There are, however, additional factors that must be considered prior to vasectomy reversal. The availability of a trained microsurgeon must certainly be taken into consideration. Without a microsurgeon skilled in both vasovasostomy as well as vasoepididymostomy available to perform the procedure, vasectomy reversal should not be undertaken and couples may be better served with sperm retrieval and IVF. Furthermore, men who have any evidence of a decline in fertility and spermatogenesis may be poor candidates for vasectomy reversal. For example, men with small, soft testes or an elevated FSH may have compromised sperm production, and additional evaluation including endocrine work-up is needed (6). The sperm production may be so impaired that the couple would require ART even if sperm returned to the ejaculate, causing a delay in achieving a pregnancy – but still requiring ART. Progressive decline in sperm production becomes more common as men age – and the population of men requiring vasectomy reversal tend to be older. Vasectomy reversal is unnecessarily invasive in subfertile males because sperm can be acquired through testicular or epididymal aspiration for the ART procedure which would have been required anyway. Furthermore, these same subfertile couples will appreciate not requiring additional contraception if the vasectomy can be maintained during and after ART. Men who had pelvic, inguinal, or prostate surgery after their vasectomy may also not be candidates for reversal because of the possibility of additional damage to the ejaculatory system which would make repair at the site of the vasectomy fruitless.

The desire for multiple children is an oft-cited reason for vasectomy reversal as a primary treatment for obstructive azoospermia. Again, however, the female partner's fertility will dictate whether this is a realistic possibility or not. Because it can take up to two years for sperm to return to the ejaculate after vasectomy reversal, at least this much time must be factored into the equation that determines a couple's likelihood of pregnancy through natural conception (7). The not inconsequential number of couples who fail their initial attempt at vasectomy reversal and require a reoperation will also have more years of waiting added to their reproductive timelines, as will the couples who have secondary stricture of the anastomosis who then require revision. Not all female partners will have the reproductive staying power to tolerate this multi-year delay, and thus a thorough understanding of her fertility potential is warranted prior to undertaking the vasectomy reversal process.

The definition of success after vasectomy reversal must be carefully evaluated when counseling patients regarding outcomes. Understandably, most authors advocate for “patency” to be the marker of success, and this number is often inappropriately quoted to patients as the “success rate” of vasectomy reversal. Although return of sperm to the ejaculate is clear evidence that the obstruction has been eliminated, and using this definition, vasectomy reversal can have impressive success rates of 44-97% (8, 9). Closer examination of the published literature and actual definition of success, however, is warranted. Couples do not request vasectomy reversal because they simply desire a return of sperm to the ejaculate; the ultimate goal is actually to have a healthy child. With this outcome in mind, vasectomy reversal is not nearly as “successful” upon re-examination of the literature as the patency rates may suggest.

Information regarding pregnancy and live birth rates from natural conception is often lacking in vasectomy outcomes papers, posing a challenge to adequately inform and counsel the couple. For example, a recent study quoted a 98% patency success rate after 1331 vasectomy reversals performed by expert microsurgeons, yet they report only 410 events of natural conception, yielding a rate of conception per vasectomy reversal of only 30.8% (410/1331); live birth rate was not addressed (10). Other studies, taken together, indicate that the rate of documented pregnancies after vasectomy reversal was only 26%, even when couples were followed for 3 years after reversal (11, 12). While many factors unrelated to the technical success of a vasectomy reversal will clearly inform this particular outcome, the drastic difference between patency rate and natural conception rate should prompt thoughtful consideration of what defines success after a vasectomy reversal.

One must realistically consider and compare these data. Vasectomy reversal has a pregnancy rate of 26% after 3 years. IVF has a pregnancy rate of 44% in each cycle, with cycles repeatable within 2 months. The cumulative pregnancy rate within 6 months will easily be more than 90%. With this frank comparison of outocome data, why even consider surgical vasectomy reversal?

Although the urge to satisfy a couple's request for a vasectomy reversal is understandable, blindly performing the procedure first without the appropriate evaluation or consideration of the option of sperm retrieval/IVF is not appropriate. For the male fertility expert counseling a couple interested in fertility after vasectomy, sperm retrieval with IVF is the preferred option. Even if vasectomy reversal is initially attempted, the vast majority of couples will require IVF.
